# Inhibition of citric acid- and capsaicin-induced cough by novel TRPV-1 antagonist, V112220, in guinea-pig

**DOI:** 10.1186/1745-9974-3-10

**Published:** 2007-12-23

**Authors:** Sum Yee Leung, Akio Niimi, Alison S Williams, Puneeta Nath, F-Xavier Blanc, Q Thai Dinh, K Fan Chung

**Affiliations:** 1 Thoracic medicine, National Heart & Lung Institute, Imperial College, London, UK; 2 Department of respiratory medicine, Graduate school of medicine, Kyoto University, Japan; 3 Department of internal medicine, Charite-Universitatsmedizin Berlin, Berlin, Germany; 4 Department of respiratory medicine, Chang Gung Memorial Hospital, Kaohsiung medical centre, Taiwan

## Abstract

**Background:**

Cough reflex can be induced by the pepper extract capsaicin and by low pH in guinea-pig airways. Transient receptor potential vanniloid-1 (TPRV-1) is expressed in the sensory and afferent nerve fibres in airways.

**Objective:**

We hypothesized that a novel pyridazinylpiperazine analog TPRV-1 inhibitor can effectively reduce cough reflex stimulated by citric acid and capsaicin.

**Methods:**

Guinea pigs were injected with specific TPRV-1 inhibitor, V112220, a pyridazinylpiperazine analog of N-(4-tertiarybutylphenyl)-4-(3-chloropyridin-2-yl) tetrahydropyrazine-1(2H)-carbox-amide (BCTC) (3 mg/kg) intra-peritoneally. One hour before cough response assessment. Coughs were recorded using a recorder system that identified cough sound and accompanying expiratory flows, distinct from sneezes. Guinea-pigs exposed to citric acid (0.4 M) and to capsaicin (10^-4^M) aerosols, in succession separately by 2 hours.

**Results:**

V112220 significantly inhibited the number of coughs induced by citric acid (73 ± 11%, p < 0.01) and capsaicin (70 ± 9.4%, p < 0.05) compared to vehicle control.

**Conclusion:**

A novel pyridazinylpiperazine analog TPRV-1 inhibitor can inhibit the cough reflex, induced by both low pH and capsaicin, suggesting that it could be clinically beneficial in treatment of cough.

## Introduction

Capsaicin is a potent tussive agent in most species including humans. It activates a capsaicin receptor, transient receptor potential vanilloid-1 (TRPV-1), which is a polymodal ion channel [1] that is activated by stimuli other than capsaicin such as, heat, acid [2] and endogenous compounds such as anandamide, bradykinin and endocannabinoids [1,3,4]. Acidification of the airway in guinea-pig also activates A-δ fibres and vagal C-fibre nerves, partly through activation of TRPV-1 [5,6]. TRPV-1 expression has been found in epithelial nerves in guinea-pig and in humans [7-9]; in chronic cough patients, the expression of TRPV-1 in epithelial nerves is enhanced [7].

Several antagonists of TRPV-1 have now been described [10]. Capsazepine is one of the first antagonists described, and blocks cough induced by capsaicin and citric acid [11-13]. In addition, other antagonists such as iodo-resiniferatoxin and BCTC have also been shown to reduce capsaicin and citric acid cough in guinea-pigs [14,15]. We investigated the effect of a novel and more selective TRPV-1 antagonist [16-18], V112220, on cough induced by capsaicin and citric acid in the conscious guinea-pig.

## Materials and methods

The protocols were approved by the Imperial College BioSciences Group and performed under a Project License from the British Home Office, UK, under the Animals (Scientific Procedures) Act 1986.

### Animals

Pathogen free Male Hartley guinea pigs (600 – 700 g) were used for the study. Animals were screened one week before the in vivo cough examination.

### Reagents

Materials used in the study including: V112220, a selective TRPV1 antagonist (Purdue Pharma, Ardsley, New York); vehicle, 20% hydroxypropyl-β cyclodextrin (Sigma, Dorset, UK); Procaterol hydrochloride (Sigma, Dorset, UK); Citric Acid (Sigma, Dorset, UK) and Capsaicin (Sigma, Dorset, UK).

### Pre-screening of animals

Conscious guinea pigs were pre-screened to assess their cough response to 0.4 M citric acid one week before the cough study with V112220 or diluent. Low responders (number of coughs < 3) and high responders (number of coughs > 20) were excluded from the study. After pre-screening, animals were allocated into 3 different groups, the control group (n = 4) and two treatment groups (either with V112220 or vehicle, n = 5).

### In vivo cough measurements

Conscious animals were placed in a 4 L plethysmograph which was equipped with an internal microphone and a pressure transducer, and were connected to a Amplifier Interface Unit series pre-amplifier (EMMS, Hants, UK). Aerosols were generated with an ultrasonic nebuliser (DeVilbiss, London, UK) which was connected to a Basic Flow Supplier AIR 200 (EMMS, Hants, UK). Airflow was set at 8 L/min. Coughs were detected in three ways: via the microphone, via the pressure transducer and by observing the guinea-pig behaviour which was also captured with an external camera. Data acquisition was performed with the eDacq (EMMS, Hants, UK) acquisition software.

### Protocol

One week following screening, guinea pigs in the treatment groups were injected with either 1 ml of vehicle or 3 mg/kg V112220 intra-peritoneally (i.p.) 1 hour before cough response assessment. Each guinea pig received 0.1 mg/kg procaterol hydrochloride i.p. injection 10 minutes prior to each cough assessment in order to minimise bronchoconstriction. For cough assessment, animals were exposed to 0.4 M citric acid for 10 min and a 10 min cough response was recorded. Two hours following citric acid inhalation, the same animal was exposed to 10^-4 ^M Capsaicin for 10 min and the cough response was assessed.

### Data analysis

Data were recorded as number of coughs per 10 min assessment. Cough numbers of individual animal were compared among pre-screening, following citric acid inhalation and following capsaicin inhalation. Data from the treatment groups were compared with the control group. Mean values were statistically analyzed by one-way analysis of variance (ANOVA) to evaluate significant differences between groups. Values are expressed as means and 95%CI, with p < 0.05 being considered significant.

## Results

### Pre-screening of animals

**Figure 1:**
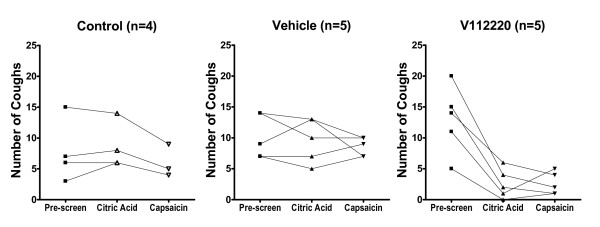
Number of coughs per 10 min in conscious guinea-pigs on pre-screen, following exposure to citric acid and to capsaicin. Left panel shows the response in the control group, the central panel the response from vehicle-treated group, and the right panel the effect of treatment with V112220.

Guinea-pigs (n = 24) were pre-screened regarding their cough response with citric acid. Ten guinea-pigs (8 low and 2 high cough responders) were excluded and subsequently, fourteen guinea-pigs were divided into 3 groups. No significant difference in baseline cough response was noted among the 3 groups of guinea pigs. Figure 1 shows the number of coughs in the 3 different groups for each guinea-pig and the number of coughs following exposure to citric acid and capsaicin at a later date either after no treatment (control) or after vehicle or after V112220 treatment.

### Effect of V112220

**Figure 2:**
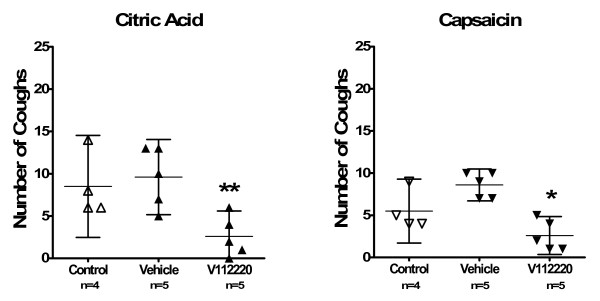
Number of coughs following citric acid or capsaicin exposure. Left panel show results from citric acid exposure while the right panel the results from capsaicin exposure. Data shown as mean ± 95% CI (*, ** p < 0.05 and 0.01 compared to vehicle treatment).

Figure 2 shows the mean cough number with 95% CI for the 3 groups of guinea-pigs for control, vehicle- and V112220-treated group. Vehicle treatment did not significantly change the number of coughs induced by either citric acid or capsaicin exposure. V112220 treatment (mean ± SEM: 2.6 ± 1.1; -0.4 to 5.6 coughs/10 min, p < 0.01) significantly reduced the number of coughs induced by citric acid compared to vehicle treatment (9.6 ± 1.6; 5.2 to 14.0 cough/10 min). V112220 treatment (2.6 ± 0.8; 0.3 to 4.9 coughs/10 min, p < 0.05) also significantly decreased the number of coughs compared to vehicle treatment (8.6 ± 0.7; 6.7 to 10.5 cough/10 min) for capsaicin. V112220 reduced citric acid-induced cough response by 73 ± 11% compared to vehicle treatment whereas capsaicin-induced cough response was reduced by 70 ± 9.4%.

## Discussion

Our study demonstrated that blockade of the TRPV-1 receptors with a selective inhibitor, V112220, which is a pyridazinylpiperazine derivative, effectively decreased by 70% coughs evoked by citric acid or capsaicin aerosol exposure in the guinea pig. This is in agreement with a previous study using the earlier TRPV-1 antagonist, capsazepine, which inhibited coughs induced by citric acid or capsaicin but not coughs induced by 7% hypertonic saline solution[12]. In addition, there have been other studies with other TRPV-1 antagonists such as iodo-resiniferatoxin and BCTC that have shown inhibition of cough induced by citric acid and capsaicin in the guinea-pig [14,15].

Recently, 4-(2-pyridyl)piperazine-1-carboxamide analogues as potent TRPV-1 antagonists have been developed [19]. N-(4-tertiarybutylphenyl)-4-(3-chloropyridin-2-yl)tetrahydropyrazine-1(2H)-carboxamide (BCTC), a member of that new chemical series, was a highly potent TRPV-1 antagonist that effectively reverses the behavioral effects of inflammatory and neuropathic pain in rats [16,17,19] but is poor in metabolic stability, short half-life, aqueous solubility, and in oral bioavailability [18]. Nevertheless, BCTC when administered intraperitoneally (30 mg/kg) one hour before capsaicin cough challenge caused an inhibition of capsaicin cough by 65% maximally [15]. A newer series of pyridazinylpiperazine compounds with improved pharmaceutical and pharmacological properties of BCTC was developed leading to V112220 which we used in this study. V112220 is similar to V113886, another pyridazinylpiperazine derivative of BCTC [18]. The plasma half-life of this compound after administration intravenously or by gavage is reported to be around 6 hours in the rat [18]. For this reason, we performed capsaicin challenge after citric acid challenge on the basis that the compound would still maintain significant plasma levels for many hours after dosing. The degree of inhibition of capsaicin- and citric acid-cough by V112220 we observed was similar with 70% reduction of the induced cough. However, capsaicin- and citric acid-cough were not completely inhibited by V112220. The incomplete inhibition, particularly of capsaicin-induced cough may indicate that higher dose of V112220 may be needed for complete inhibition, since cough response induced by capsaicin is presumed to be entirely mediated by TRPV-1. However, further studies with higher doses will be needed to answer this issue.

TRPV1 is sensitive to vanilloid molecules, including capsaicin. It can be activated by low extracellular pH [2,6,20,21], and by the endocannabionid, anandamide [22], lipoxygenase metabolites [23] and N-arachidonoyl-dopamine [24], and also by a change in temperature [25]. TRPV1 is highly expressed in a subset of primary sensory neurons of the trigeminal, vagal and dorsal root ganglia with C- and A-δ fibres [9]. These receptors are polymodal nociceptors. TRPV1 excites terminals of primary sensory neurons and causes the initiation of action potentials of reflex responses, such as cough in airways [26]. It may also cause a series of neurogenic inflammation via antidromic conduction of action potential to collateral nerve fibres [26]. Capsaicin is one of the most tussigenic stimuli available in conscious animals and humans, and TRPV1 has been identified as a possible component of the cough receptor in guinea pigs and humans [27]. Inflammatory stimuli such as prostaglandins, bradykinin, and nerve growth factor may upregulate the expression and function of TRPV-1 [28-30]. Chronic airway inflammation such as in asthma or COPD may increase the sensitivity of TRPV-1 to its agonists and trigger the cough reflex [27]. The expression of TRPV-1 in the epithelial airway nerves of patients with chronic persistent cough of diverse causes and with an enhanced capsaicin cough response has a 3-fold increase of TRPV-1 expression [7]. TRPV-1 receptors may therefore contribute to the enhanced cough reflex and the cough response in chronic persistent cough

Chronic persistent cough is a clinical problem, since antitussives available to control cough are often not effective [31]. More potent antitussives are needed. TRPV-1 antagonists may represent a potential class of antitussives that could be useful in the control of chronic persistent cough.

## Authors' contributions

SYL carried out the drugs administration and cough measurements, and performed the statistical analysis. AN helped in the design of the study. ASW & PN participated in animal maintenance. F-XB & QTD assisted in cough measurement, and drugs preparation. KFC conceived of the study, and participated in its coordination. All authors read and approved the final manuscript.
